# Association of first-trimester exposure to Kampo medicines containing *Prunus persica* kernel with preterm birth and major congenital malformations: a Japanese database study

**DOI:** 10.3389/fphar.2025.1562724

**Published:** 2025-08-22

**Authors:** Satoko Suzuki, Ryutaro Arita, Taku Obara, Tomofumi Ishikawa, Tadaharu Kunitoki, Takamasa Sakai, Aoi Noda, Genki Shinoda, Mami Ishikuro, Masatsugu Orui, Shinichi Kuriyama, Minoru Ohsawa, Ken Haneda, Nariyasu Mano, Akiko Kikuchi, Shin Takayama, Tadashi Ishii

**Affiliations:** ^1^ Department of Education and Support for Regional Medicine (General and Kampo Medicine), Tohoku University Hospital, Sendai, Japan; ^2^ Department of Kampo and Integrative Medicine, Graduate School of Medicine, Tohoku University, Sendai, Japan; ^3^ Department of Pharmaceutical Sciences, Tohoku University Hospital, Sendai, Japan; ^4^ Division of Molecular Epidemiology, Department of Preventive Medicine and Epidemiology, Tohoku Medical Megabank Organization, Tohoku University, Sendai, Japan; ^5^ Laboratory of Biomolecule and Pathophysiological Chemistry, Graduate School of Pharmaceutical Sciences, Tohoku University, Sendai, Japan; ^6^ Division of Clinical Pharmacology and Therapeutics, Tohoku University Graduate School of Pharmaceutical Sciences, Sendai, Japan; ^7^ Drug Informatics, Faculty of Pharmacy, Meijo University, Nagoya, Japan; ^8^ Department of Obstetrics and Gynecology, Tohoku University Hospital, Sendai, Japan

**Keywords:** database, pregnancy, malformation, preterm birth, Prunus persica kernel, Kampo medicine, traditional medicine

## Abstract

**Introduction:**

Traditional Japanese (Kampo) medicine containing *Prunus persica* kernel (KPK) is prescribed for treating menstrual- and pregnancy-related symptoms. However, no safety information is available regarding its use in pregnant women. In this study, we examined the associations of KPK prescriptions during the first trimester of pregnancy with preterm births and major congenital malformations (MCMs) in newborns.

**Methods:**

From a large-scale Japanese health insurance claims database, we included pregnant women enrolled with the same healthcare insurer from 3 months before pregnancy to the date of delivery, who gave birth between 2010 and 2019, and whose data were linked to their infants. We then selected pregnant women who were prescribed KPK during the first trimester as the exposure group, and those who were prescribed tokishakuyakusan (TSS), commonly used for pregnancy-related symptoms, during the same period as controls. The association between KPK prescriptions and preterm birth or MCM among infants was examined using a multivariate logistic regression analysis.

**Results:**

Of the 75,398 infants, TSS and KPK was prescribed to 2,548 (3.38%) and 283 (0.38%) women during the first trimester, respectively. In the TSS group, 311 of 2,491 infants (12.5%) experienced preterm births, whereas 40 of 283 infants (14.1%) in the KPK group experienced preterm births. The risk of preterm birth in the KPK group was not significantly different from that in the TSS group (adjusted risk ratio, 1.122; 95% confidence interval, 0.827–1.521). In the TSS group, 157 of 2,491 infants (6.3%) had MCMs, whereas 15 of 283 infants (5.3%) in the KPK group had MCMs. There was no significant difference in the incidence of MCM in the first year after birth between infants in the KPK and TSS groups (adjusted odds ratio, 0.820; 95% confidence interval, 0.475–1.415).

**Conclusion:**

There was no significant difference in the risk of preterm birth or MCMs between pregnant women prescribed KPK and those prescribed TSS during the first trimester.

## 1 Introduction

Pregnant women experience a variety of pregnancy-related symptoms. In the first trimester, nausea, vomiting, pelvic cavity pain, and back pain are the most common symptoms, with 88% of women reporting multiple symptoms (Lutterodt et al., 2019). However, the use of medications during pregnancy requires careful consideration because of the potential for adverse maternal and fetal outcomes. Given the limitations in using conventional medications during pregnancy due to potential risks, alternative treatments such as Kampo medicine are often considered.

Japanese traditional (Kampo) medicine is derived from Chinese herbal medicines and was uniquely developed in Japan (Motoo et al., 2011). Kampo formulas are composed of multiple natural herbs and are used to treat pregnancy-related symptoms by the national health insurance system in Japan (Yoshino et al., 2023). Tokishakuyakusan (TSS) is the most commonly used Kampo formula for treating pregnancy-related symptoms, such as edema, headache, and dizziness, and for supporting fetal development, so its continuous use during pregnancy has empirically been a common practice. Our previous study investigated 8% of pregnant women prescribed TSS in a Japanese large-scale claims database. (Suzuki et al., 2021).

*Prunus persica* kernel (PK) is also a natural herb used to treat gynecological diseases. The current Japanese Pharmacopoeia classifies PK as seeds of *P. persica* Batsch or *P. persica* Batsch var. *davidiana* Maximowicz (*Rosaceae*), which contains no less than 1.2% amygdalin (The Ministry of HealthLabour and Welfare, 2021). PK exerts multiple pharmacological effects, including anti-inflammatory, antioxidant, and immune regulatory effects, and is clinically used not only for gynecological diseases but also for cardiovascular, anal, digestive, and orthopedic conditions (Nowicka and Wojdyło, 2019; Liu et al., 2024).

There is limited discussion on the lack of modern scientific evidence regarding PK’s contraindication during pregnancy. Package inserts of Kampo formulas containing PK (KPK) indicate that their use is not recommended for pregnant women or women who may possibly be pregnant and that PK may cause preterm birth or abortion (Tsumura and Co, 2007), though it is not prohibited. This recommendation is based on classical books on traditional Chinese medicine. For example, The Compendium of Materia Medica (Honzokomoku in Japanese, Bencao Gangmu in Chinese), published in the 16th century, describes PK as a natural herb prohibited during pregnancy. A literature search revealed one article in Chinese that reported the teratogenic effects of PK; however, detailed information on the study design and results is lacking (Xi et al., 2013). Despite traditional contraindications, there is a notable absence of modern clinical studies evaluating the safety of PK during pregnancy. Given the ongoing use of KPKs among pregnant women and the lack of empirical safety data, it is imperative to investigate the potential risks associated with their use.

Our previous research investigating large-scale claims data showed that KPKs (e.g., keishibukuryogan, tokakujokito, and junchoto) were used by pregnant women (Suzuki et al., 2021). The prescription of KPK might occur when physicians have concluded that the benefits are greater than the potential risks. However, the safety of KPK in pregnant women and infants has not been investigated. The aim of this study was to investigate the associations of first-trimester exposure to KPK with preterm birth and major congenital malformations (MCMs) in infants using a large claims database.

## 2 Methods

### 2.1 Database

Data were obtained from JMDC Inc. (Tokyo, Japan) (Kimura et al., 2010). This database is anonymous and privacy-protected, and includes details such as sex, birth year, and month. We extracted insurance claims information, diagnoses defined by the International Classification of Diseases, 10th Revision (ICD-10) codes, drug prescriptions covered by health insurance at hospitals, including outpatient and inpatient care, dispensing at pharmacies, and procedures including operations. The Institutional Review Board of Tohoku University School of Medicine approved this study on 22 January 2024 (registration number: 2023-1-808). Informed consent was not required because the obtained data were de-identified.

### 2.2 Study population

**FIGURE 1:**
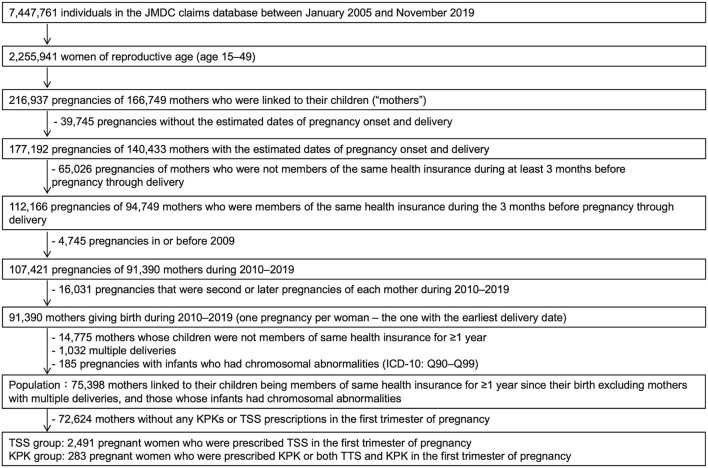
Flow chart illustrating the selection of the study population for data analysis. ICD, international classification of disease; KPK, Kampo medicines containing *Prunus persica* kernel; TSS, tokishakuyakusan.

We used the dataset available on 8 May 2020, for this study, which included 7,447,761 men and women covered by health insurance between January 2005 and November 2019, similar to our previous study (Suzuki et al., 2023). The insured individuals had anonymized family and personal identification numbers. Therefore, we identified mother-child relationships among individuals if data regarding their newborns were entered by an identical health insurer with matching family identification numbers. In addition, this dataset allowed us to identify the birth year and month (information regarding the date of birth was not included). The study population included mothers who met the following eligibility criteria: mothers who were linked to their infants whose birth month was in accordance with the month of enrollment in the health insurance, mothers who continued to be covered under the same health insurance from 3 months prior to pregnancy until delivery, and mothers whose dates of pregnancy onset and delivery were estimable. Figure 1 shows the flowchart of the patient selection process. The database contained a limited number of women who gave birth before 2009; thus, we included only those women who became pregnant between 2010 and 2019. Only one pregnancy per woman, which was the first pregnancy estimated in the database, was included. Furthermore, to investigate the risk of preterm birth or MCM due to exposure to KPK in first-trimester pregnancy, mothers whose newborns were covered by the same health insurance during their birth month for more than a year after birth were included. Conversely, mothers with multiple gestations were excluded because the risk of congenital disabilities increases with multiple births (Zhang et al., 2011). Mothers whose infants had chromosomal abnormalities (ICD-10 codes Q90–Q99) (Huybrechts et al., 2019) were also excluded because they were not associated with exposure, and their inclusion would reduce the detection of exposure-related risk.

### 2.3 Pregnancy onset and delivery date estimations

As no data on pregnancy onset or delivery date were obtained from this database, we used previously reported estimation methods (Ishikawa et al., 2018; Ishikawa et al., 2019; Ishikawa et al., 2020; Suzuki et al., 2021; Ishikawa et al., 2022; Ishikawa et al., 2023; Suzuki et al., 2023). The pregnancy onset date was calculated by subtracting the gestational age associated with the diagnosis from the date of the disease diagnosis (Ishikawa et al., 2018; Ishikawa et al., 2019; Ishikawa et al., 2020; Suzuki et al., 2021; Ishikawa et al., 2022; Ishikawa et al., 2023). In cases where a mother had several visits with diagnoses, the most recent gestational age was considered because a more accurate diagnosis could be made in the later stages of pregnancy (Committee on Obstetric Practicethe American Institute of Ultrasound in Medicinethe Society for Maternal-Fetal Medicine, 2017). A study based on university hospital records demonstrated that approximately 90% of the pregnancy onset dates estimated using this method fell within ±1 week of the true pregnancy onset, indicating a high accuracy (Ishikawa et al., 2018).

The delivery date was estimated from delivery-related entries and infant birth months using a previously reported algorithm (Ishikawa et al., 2019; Suzuki et al., 2021). A study based on university hospital records demonstrated that approximately 95% of the estimated delivery dates were within 1 week of the actual delivery date (Ishikawa et al., 2018). In Japan, health insurance does not always cover deliveries that do not require procedures or medication. Therefore, in cases where delivery data were unavailable, the 15th day of the month of the birth month was considered as the delivery date. Induction of labor is recommended in women with a pregnancy duration of over 294 days because of increased perinatal mortality (Minakami et al., 2014). When the difference between the estimated dates of delivery and pregnancy onset was >294 days, the gestational period was set at 294 days and pregnancy onset was subtracted from the estimated delivery date by 294 days. The first trimester was defined as the duration from pregnancy onset up to week 13 and day 6 of gestation, the second trimester as from week 14 days 0 to week 27 day 6 of gestation, and the third trimester as week 28 day 0 until delivery (Minakami et al., 2014).

### 2.4 Exposures

We set tokishakuyakusan (TSS) as a competitor drug. TSS is a Kampo medicine that does not contain PK and has been used to treat pregnancy-related symptoms, such as edema, headache, and dizziness (Morohashi et al., 2020; Nakayama et al., 2021; Suzuki et al., 2021; Toda et al., 2021). The package insert of TSS states its indications for symptoms during pregnancy (Tsumura and Co, 2014). The TSS was composed of peony root, Atractylodes lancea rhizome, Alisma rhizome, Poria sclerotium, Cnidium rhizome, and Japanese Angelica root (Supplementary Table S1). TSS has been reported to activate blood (Shimizu et al., 2022), have blood-replenishing effects (Akase et al., 2007), prevent miscarriage (Nagamatsu et al., 2018), and promote fetal growth (Li et al., 2013); therefore, TSS is used to manage various conditions in pregnant women. Our previous studies have shown that TSS is one of the frequently prescribed Kampo medicines during pregnancy (Suzuki et al., 2021; Noda et al., 2024).

The exposure drugs are KPK, which include the following eight Kampo formulations; choyoto, daiobotampito, junchoto, keishibukuryogan, keishibukuryogankayokuinin, sokeikakketsuto, tokakujokito. The composition crude drugs are listed in Supplementary Table S1. The maximum daily amount of PK are 2–5 g. The exposure group comprised pregnant women who were prescribed KPK and crude *P. persica* kernel, including pregnant women who received both TSS and KPK. We used the methods reported in a previous study to estimate the timing of exposure to these medications (Suzuki et al., 2023).

The timing of drug exposure was based on the day of dispensing. In Japan, dispensing dates are recorded on receipts primarily for outpatient prescriptions filled at external pharmacies; they are missing for inpatient prescriptions administered during hospital stays. If the dispensing date was not available in the database, the date of admission was considered for calculation. If neither the dispensing date nor the admission date could be obtained, the 15th of the month was used as the dispensing date because the month and year were listed on each claim. The exposure period was calculated as the number of days exposed to each prescription. We extracted drug exposures during the first trimester of pregnancy, which is the most critical period for organogenesis, to analyze their association with maternal and fetal outcomes.

### 2.5 Outcomes

Preterm birth was defined as an estimated pregnancy period of <37 weeks (Goldenberg et al., 2008). Supplementary Table S2 shows the MCMs definition with ICD-10 codes (Q00–Q89). Minor congenital malformations were excluded, and diagnoses of MCMs administered to Infants during the first year of life were extracted (Ishikawa et al., 2021). Additionally, MCMs were grouped separately based on individual organ systems that were affected. A study based on university hospital records demonstrated that MCMs in claims were validated against medical records, and the overall positive predictive value of MCMs was approximately 90% (Ishikawa et al., 2021).

### 2.6 Covariates

We considered the following variables as covariates: maternal age at birth; birth year; medical history of epilepsy, diabetes, and obesity; and prescription of teratogenic drugs in the first trimester of pregnancy. These covariates were defined on the basis of previous studies (Eriksson et al., 2000; Oliveira and Fett-Conte, 2013; Minakami et al., 2014; Alvarado-Terrones et al., 2018; Mbizvo et al., 2020) (Supplementary Table S3). These covariates were considered a possible determinant of exposure, outcome, or both (VanderWeele, 2019).

### 2.7 Statistical analyses

To investigate the preterm birth risk and the MCM risk exposure among the KPK group prescribed in the first trimester of pregnancy, two groups were selected from the study population: pregnant women who were prescribed TSS in the first trimester of pregnancy (control group), and those who were prescribed KPK in the first trimester of pregnancy (KPK group). Women prescribed both TSS and KPK were placed in KPK group.

Between the two groups, the risk ratios (RR) and 95% confidence intervals (CI) for preterm birth were evaluated using modified Poisson regression analysis and the odd ratios (OR) and 95% CI for MCM were evaluated using logistic regression analysis. The results were assessed after adjusting for multiple covariates. Sensitivity analysis was also performed. To analyze the risk of preterm birth, the analysis was conducted after excluding pregnant women with combined TSS and KPK prescriptions. To analyze MCM risk, the analyses were repeated after 1) including only women who did not receive a prescription for a suspected teratogenic drug in the first trimester of pregnancy, 2) including only pregnant women who had been prescribed TSS and KPK for more than 30 days during the first trimester, and 3) excluding pregnant women with combined prescriptions of TSS and KPK.

Differences were considered significant when the 95% CIs did not cross 1.0. Statistical significance was set at P < 0.05. difference. All statistical analyses were performed using SAS version 9.4 (SAS Institute Inc., Cary, NC, United States).

## 3 Results

### 3.1 Population characteristics

We identified 75,398 women who were eligible for screening in this study (Figure 1). The distribution of the methods of estimating delivery dates was as follows: 37,213 (49.4%) based on the dates of specific diagnoses and procedures for the aforementioned algorithms; 7,117 (9.4%) based on the dates of other delivery-related entries; 31,608 (41.2%) based on the 15th day of the neonatal birth month; and 1,134 (1.5%) evenly assigned 294 days as the duration of gestation.

During pregnancy, TSS and KPK was prescribed to 6,576 (8.72%) and 411 (0.55%) women, respectively (Table 1). Among the prescriptions of KPK, keishibukuryogan (203, 0.27%) was the most frequently prescribed, followed by tokakujokito (74, 0.10%), keishibukuryogankayokuinin (58, 0.08%), and junchoto (51, 0.07%). TSS and KPK was prescribed to 2,548 (3.38%) and 283 (0.38%) women during the first trimester, respectively.

**TABLE 1:** Prescription of TSS and KPK during the pregnancy.

Name of medicinal product	Total population		First trimester		Second trimester		Third trimester	
	n = 75,398		n = 75,398		n = 75,398		n = 75,224	
	n	%	n	%	n	%	n	%
Total	6,867	9.11%	2,774	3.68%	2,578	3.42%	3,697	4.91%
TSS	6,576	8.72%	2,548	3.38%	2,523	3.35%	3,611	4.80%
KPK	411	0.55%	283	0.38%	63	0.08%	112	0.15%
Keishibukuryogan	203	0.27%	140	0.19%	14	0.02%	59	0.08%
Tokakujokito	74	0.10%	54	0.07%	14	0.02%	17	0.02%
Sokeikakketsuto	13	0.02%	3	0.00%	7	0.01%	7	0.01%
Keishibukuryogankayokuinin	58	0.08%	53	0.07%	4	0.01%	4	0.01%
Junchoto	51	0.07%	23	0.03%	22	0.03%	23	0.03%
Daiobotampito	8	0.01%	5	0.01%	2	0.00%	2	0.00%
Crude *Prunus persica* kernel	14	0.02%	13	0.02%	0	0.00%	1	0.00%
Choyoto	4	0.01%	5	0.00%	0	0.00%	1	0.00%

TSS, tokishakuyakusan; KPK, kampo medicines containing *prunus persica* kernel.

### 3.2 Preterm birth risks of first-trimester exposure to KPK

Of the 75,398 infants, 8,790 (11.7%) experienced preterm births. In the TSS group, 311 of 2,491 infants (12.5%) experienced preterm births, whereas 40 of 283 infants (14.1%) in the KPK group experienced preterm births (Table 2). The risk of preterm birth in the KPK group was not significantly different from the risk in the TSS group (crude RR, 1.132; 95%CI, 0.834-1.536; adjusted RR, 1,122; 95%CI, 0.827-1.521) (Table 3). Sensitivity analysis produced a result similar to those of the primary analysis.

**TABLE 2:** Characteristics of the study population and prevalence of preterm birth and MCMs.

Variables	Total population		Women with TSS in the first trimester		Women with KPK in the first trimester	
	(n = 75,398)		(n = 2,491)		(n = 283)	
Maternal age at delivery, mean ± SD	32.3±4.6		32.9±4.6		33.2±4.6	
Caesarean section	16,710	22.2%	635	25.5%	71	25.1%
Hypertension	6,248	8.3%	56	2.2%	7	2.5%
Diabetes	6,559	8.7%	136	5.5%	19	6.7%
Obesity	735	1.0%	25	1.0%	2	0.7%
Epilepsy	508	0.7%	24	1.0%	6	2.1%
Prescription of suspected teratogenic medication in the first trimester	275	0.4%	8	0.3%	3	1.1%
Newborn sex male/female	38,611/36,787	51.2%/48.8%	1,305/1,186	52.4/47.6%	134/149	47.3/52.7%
Preterm birth	8,790	11.7%	311	12.5%	40	14.1%
MCMs overall	4,607	6.1%	157	6.3%	15	5.3%

KPK, Kampo medicines containing Prunus persica kernel.; MCM, major congenital malformation; TSS, tokishakuyakusan.

**TABLE 3:** Associations between KPK prescribed during the first trimester of pregnancy and preterm birth.

	Women with TSS in the first trimester		Women with KPK in the first trimester		Crude RR (95% CI)	Adjusted RR (95% CI)
	Number of preterm birth	Number of women	Number of preterm birth	Number of women		
Primary analysis	311	2,491	40	283	1.13 (0.83–1.54)	1.12 (0.83–1.52)
Sensitivity analysis: Excluding pregnant women with combined TSS and KPK prescriptions	311	2,491	30	226	1.06 (0.75–1.51)	1.07 (0.76–1.51)

KPK, Kampo medicines containing Prunus persica kernel.; RR, risk ratio; CI, confidence interval; TSS, tokishakuyakusan.

### 3.3 MCM risks of first-trimester exposure to KPK

Of 75,398 infants, 4,607 (6.1%) were diagnosed with MCMs within the first year after birth. In the TSS group, 157 of 2,491 infants (6.3%) had MCMs, whereas 15 of 283 infants (5.3%) in the KPK group had MCMs (Table 2). There was no significant difference in terms of the assignment of MCM in the first year after birth between the births among pregnant women in the TSS and KPK groups (crude OR, 0.832; 95% CI, 0.483–1.434; adjusted OR, 0.820; 95% CI, 0.475–1.1415). The sensitivity analyses supported the main analysis (Table 4).

**TABLE 4:** Associations between KPK prescribed during the first trimester of pregnancy and MCM.

	Women with TSS in the first trimester		Women with KPK in the first trimester		Crude OR (95% CI)	Adjusted OR (95% CI)
	Number of MCMs	Number of women	Number of MCMs	Number of women		
Primary analysis	157	2,491	15	283	0.83 (0.48-1.43)	0.820 (0.48-1.42)
Sensitivity analysis 1 Including only pregnant women who did not receive a prescription for a suspected teratogenic drug in the first trimester of pregnancy	157	2,483	15	280	0.84 (0.49-1.45)	0.820 (0.48-1.42)
Sensitivity analysis 2 Including only pregnant women who had been prescribed TSS and KPK for more than 30 days during the first trimester	59	862	5	132	0.54 (0.21-1.36)	0.550 (0.22-1.40)
Sensitivity analysis 3 Excluding pregnant women with combined prescriptions of TSS and KPK	157	2,491	10	226	0.69 (0.36-1.32)	0.682 (0.35-1.32)

KPK, Kampo medicines containing Prunus persica kernel.; MCM, major congenital malformation; OR, odds ratio; CI, confidence interval; TSS, tokishakuyakusan.

## 4 Discussion

In this study, using a large-scale clinical database, no significant association was found between the frequency of MCMs in the infants of pregnant women prescribed KPK and TSS during the first trimester of pregnancy. Furthermore, there was no association between the frequency of preterm delivery in pregnant women prescribed KPK and those prescribed TSS during the first trimester of pregnancy. These novel findings suggest that KPK can be safely administered during the first trimester of pregnancy.

While previous article has reported teratogenic and abortifacient effects of PK according to literature search (Xi et al., 2013), our findings did not observe significant association with MCMs, suggesting the opposite indication. While literature reviews often base their findings on animal studies or classical texts, which may involve different administration dosage or periods than our research, our findings suggest that the risk of using KPK during the first trimester warrants re-evaluation. Furthermore, the association with other pregnancy-related outcomes beyond MCM and miscarriage needs to be clarified through future research.

KPK is known to promote uterine contractions and has been used to expel the retained placenta after delivery. Two cases have been reported in the literature where retained placenta was successfully expelled without surgery using traditional medicines containing PK (Nishida et al., 2011; Huang et al., 2021). A recent meta-analysis from China revealed that supplementation with Shenghua decoction, which contains PK, was associated with a higher complete abortion rate in women with early medical abortion and was associated with no adverse events (Li et al., 2022). The use of PK during pregnancy has not been recommended in classical, traditional Chinese medicine books or modern Kampo formula package inserts because of this potential risk of preterm birth. However, our findings challenge the conventional wisdom that PK should be avoided during pregnancy due to its potential risks.

Previous studies using traditional medicine database do not describe PK or KPK. A database study from Taiwan reported that 20%–33% of pregnant women used traditional medicine, with greater tendency to use it among patients with threatened abortion (Yeh et al., 2009; Chuang et al., 2009; Wen et al., 2020). Furthermore, while Coptis rhizome and An-Tai-Yin use during the first trimester was associated with an increased risk of some congenital malformations (Chuang, et al., 2006.), no papers mentioned the risk associated with PK. We hope that the results of this study will contribute to the choice of KPK for use during pregnancy.

According to the traditional framework of Kampo medicine, PK is used to address the *blood stasis pattern* (TM1). This condition is clinically characterized by features such as darkened complexion, localized bluish or purplish lumps, localized pain, bleeding with dark-colored blood and clots, a purple or spotted tongue, purple lips, and a wiry, firm, or choppy pulse. These manifestations are commonly associated with various menstrual disorders (WHO, 2024). Kampo prescriptions, particularly KPKs, are often administered to women with infertility attributed to blood-related abnormalities (Yoshida-Komiya et al., 2021). They are typically recommended for women of reproductive age who present with dysmenorrhea in conjunction with evidence of blood disorders. Consequently, it is critical to establish robust safety parameters for the use of KPK during pregnancy to alleviate concerns among pregnant women and their families who are prescribed these treatments.

We conducted several sensitivity analyses. Almost of all analyses revealed similar results to those in primary analyses. In the sensitivity analysis two for MCM risk, the point estimate of OR in KPK group was 0.536. The reason for this may be that the sample size was reduced by limiting women prescribed laxatives for more than 30 days during the first trimester of pregnancy, resulting in a lower prevalence of MCM, especially in the KPK group. Therefore, it should be noted that the prevalence is not necessarily higher in the TSS group.

This study is subject to several limitations that warrant discussion. First, the prescription of KPK in the first trimester was smaller than that of TSS (0.38% and 3.38%, respectively). Despite the possibility that the infrequent prescription of KPK to pregnant women indicates adherence to non-recommendations, the limited exposure numbers might have hindered a robust calculation of the actual risk of adverse events. Second, there is less evidence of the safety of TSS, the competitor drug in this study. TSS has been utilized for pregnancy-related symptoms for hundreds of years and was prescribed to 8% of pregnant women (Suzuki et al., 2021). Its tolerability has historically not been a significant concern. A recent basic study showed the safety of TSS on the hatching tare from embryo and morphology of the juvenile of *Danio rerio* (Luo et al., 2024). Further basic and clinical studies to elucidate the safety of TSS during pregnancy are warranted. Third, Kampo medicine prescriptions may not accurately reflect the actual consumption of the prescribed substances. If patients did not adhere to the prescribed regimen, the findings might be influenced by exposure misclassification bias. However, the utilization of prescription data minimizes recall bias that could arise from reliance on self-reported information. the safety of TSS is historically assured, though scientific evidence is limited. Forth, TSS and KPK are available at pharmacies as over-the-counter drugs; however, data on those purchased outside health insurance could not be obtained. Fifth, we could not obtain data on smoking habit or folic acid supplementation which are associated with MCMs (Harris et al., 2017), because the database used in this study was based on insurance claim data, and it did not include this information. Sixth, our previous report has shown that in the case of preterm births, the percentage of births within 7 days of the gold standard birth date estimated from office data is about 85% (Ishikawa et al., 2021), which may cause slight misclassification. There are no reports on positive predictive value (PPV) with preterm birth as an outcome, and future studies on the accuracy of preterm birth estimation based on Japanese claims data and other data are desirable. Seventh, while this study evaluated MCMs and preterm births, it excluded data on abortions and stillbirths due to the study design prioritizing linkage between mother and child. The lack of research on the effects of KPK on stillbirths and miscarriages presents a gap that may contribute to underestimation of its harm. Additionally, the effect of KPK exposure after the second trimester of pregnancy on preterm births should also be evaluated. However, we first analyzed KPK during the first trimester of pregnancy in the same population as the MCM assessment, with priority given to obtaining bias-free results. Future studies should adopt alternative designs to address this limitation and assess the impact of KPK on these outcomes. Lastly, the maximum daily dosage of PK in Japanese Kampo extract formulations is relatively low, capped at 5 g (Supplementary Table S1). More amount of PK could be consumed when multiple PKs are prescribed, or when decoction formula containing PK is used for severe disease conditions. Further research is essential to investigate whether higher dosages of PK during pregnancy might influence the incidence of MCM or preterm births in infants, because higher dosage are generally associated with an increased risk of adverse effects. Despite the research limitations described, the present study is the first study based on a quantitative analysis rather than a literature search, using a large-scale clinical database, of the association between KPK during the first trimester of pregnancy and risk of MCMs and preterm births.

## 5 Conclusion

This study found no significant differences in the risk of preterm birth or MCMs between pregnancies exposed to KPK and those exposed to TSS. These findings suggest that the use of KPK during the first trimester of pregnancy does not pose additional risks for these outcomes compared to TSS prescriptions, providing important preliminary evidence to inform the safe use of KPK in clinical practice. Further research is needed to validate these findings and explore their implications for maternal and fetal health.

## Data Availability

The raw data supporting the conclusions of this article will be made available by the authors, without undue reservation.
