# Supplementary material for: Association of first-trimester exposure to Kampo medicines containing Prunus persica kernel with preterm birth and major congenital malformations: a Japanese database study
Source: Front Pharmacol. 2025 Aug 22;16:1562724. doi: 10.3389/fphar.2025.1562724 (PMC12411776; doi:10.3389/fphar.2025.1562724)
Supplement: Supplementary file 1 [file Table1.docx]

Supplementary Material

# Supplementary Tables

Supplemental Table 1. Kampo medicines containing *Prunus persica* kernel and those composition.

| **Kampo medicines** | | **Composition** | **Maximum daily mount of PK (g)** |
| --- | --- | --- | --- |
| **Competitor** | | |  |
|  | Tokishakuyakusan  (TSS) | •Root of *Paeonia lactiflora*  •Rhizome of 1) *Atractylodes japonica,* 2) *Atractylodes macrocephala,* 3) *Atractylodes lancea,* or 4) *Atractylodes chinensis*  •Tuber of *Alisma orientale*  •Sclerotium of *Wolfiporia cocos*  •Rhizome of *Cnidium officinale*  •Root of *Angelica acutiloba* or *Angelica acutiloba* var. *sugiyamae* | 0 |
| **Kampo medicine containing *Prunus persica* kernel (KPK)** | | |  |
|  | Choyoto | •Seed of *Coix lachryma-jobi*  *•*Seed of *1) Benincasa cerifera or 2) Benincasa cerifera* forma *emarginata*  *•*Seed of *Prunus persica* or *Prunus persica* var*. davidiana*  *•*Sclerotium of *Wolfiporia cocos*  *•*Root bark of *Paeonia suffruticosa* | 5 |
|  | Daiobotampito | *•*Seed of *1) Benincasa cerifera or 2) Benincasa cerifera* forma *emarginata*  *•*Seed of *Prunus persica* or *Prunus persica* var*. davidiana*  *•*Root bark of *Paeonia suffruticosa*  *•*Rhizome of *Rheum palmatum, Rheum tanguticum, Rheum officinale, Rheum coreanum* or their interspecific hybrids  *•*Sodium sulfate (Na2SO4) containing no water of crystallization | 4 |
|  | Junchoto | •Root of *Rehmannia glutinosa* var. *purpurea* or *Rehmannia glutinosa*  •Root of *Angelica acutiloba* or *Angelica acutiloba* var. *sugiyamae*  *•*Root of *Scutellaria baicalensis*  •Immature fruit or the fruit cut crosswise of *Citrus aurantium* var. *daidai, Citrus aurantium* or *Citrus natsudaidai*  *•*Seed of *Prunus armeniaca, Prunus armeniaca* var. *ansu,* or *Prunus sibirica*  •Bark of the trunk of *Magnolia obovate, Magnolia officinalis* or *Magnolia officinalis*  *•*Rhizome of *Rheum palmatum, Rheum tanguticum, Rheum officinale, Rheum coreanum* or their interspecific hybrids  *•*Seed of *Prunus persica* or *Prunus persica* var*. davidiana*  •Fruit of *Cannabis sativa*  *•*Root and stolon, with (unpeeled) or without (peeled) the periderm, of *Glycyrrhiza uralensis* or *Glycyrrhiza glabra* | 2 |
|  | Keishibukuryogan | •Bark of the trunk of *Cinnamomum cassia*  •Root of *Paeonia lactiflora*  *•*Seed of *Prunus persica* or *Prunus persica* var*. davidiana*  *•*Sclerotium of *Wolfiporia cocos*  *•*Root bark of *Paeonia suffruticosa* | 3-4 |
|  | Keishibukuryogan-kayokuinin | •Seed of *Coix lachryma-jobi*  •Bark of the trunk of *Cinnamomum cassia*  •Root of *Paeonia lactiflora*  *•*Seed of *Prunus persica* or *Prunus persica* var*. davidiana*  *•*Sclerotium of *Wolfiporia cocos*  *•*Root bark of *Paeonia suffruticosa* | 4 |
|  | Sokeikakketsuto | •Root of *Paeonia lactiflora*  •Root of *Rehmannia glutinosa* var. *purpurea* or *Rehmannia glutinosa*  •Rhizome of *Cnidium officinale*  •Rhizome of 1) *Atractylodes japonica,* 2) *Atractylodes macrocephala,* 3) *Atractylodes lancea,* or 4) *Atractylodes chinensis*  •Root of *Angelica acutiloba* or *Angelica acutiloba* var. *sugiyamae*  *•*Seed of *Prunus persica* or *Prunus persica* var*. davidiana*  *•*Sclerotium of *Wolfiporia cocos*  •Root with rhizome of *Clematis mandshurica, Clematis chinensis,* or *Clematis hexapetala*  •Root of *Notopterygium incisum* or *Notopterygium forbesii*  *•*Root of *Achyranthes bidentata* or *Achyranthes fauriei*  *•*Pericarp of the ripe fruit of *Citrus unshiu* or *Citrus reticulata*  *•*Climbing stem and rhizome of *Sinomenium acutum*  *•*Root and rhizome of *Saposhnikovia divaricate*  *•*Root and rhizome of *Gentiana scabra, Gentiana manshurica* or *Gentiana trifloral*  *•*Root and stolon, with (unpeeled) or without (peeled) the periderm, of *Glycyrrhiza uralensis* or *Glycyrrhiza glabra*  *•*Root of *Angelica dahurica*  *•*Rhizome, with (unpeeled) or without (peeled) the periderm, of *Zingiber officinale* | 2 |
|  | Tokakujokito | *•*Seed of *Prunus persica* or *Prunus persica* var*. davidiana*  •Bark of the trunk of *Cinnamomum cassia*  *•*Rhizome of *Rheum palmatum, Rheum tanguticum, Rheum officinale, Rheum coreanum* or their interspecific hybrids  *•*Root and stolon, with (unpeeled) or without (peeled) the periderm, of *Glycyrrhiza uralensis* or *Glycyrrhiza glabra*  *•*Sodium sulfate (Na2SO4) containing no water of crystallization | 5 |
| Crude *Prunus persica* kernel | | *•*Seed of *Prunus persica* or *Prunus persica* var*. davidiana* |  |

Supplemental Table 2. List of the ICD-10 codes selected as MCMs

| MCM | ICD-10 codes |
| --- | --- |
| MCMs of the nervous system | Q00-Q07 |
| MCMs of the eyes, ears, face, and neck | Q10-Q18（Q10, Q162, Q17–Q182, Q184–Q189 excepted） |
| MCMs of the circulatory system | Q20-Q28（Q250, Q270 excepted） |
| MCMs of the respiratory system | Q30-Q34 |
| Cleft lip and cleft palate | Q35-Q37 |
| MCMs of the digestive system | Q38-Q45（Q381 excepted） |
| MCMs of the genital organs | Q50-Q56（Q515, Q516, Q520–Q527, Q53 excepted） |
| MCMs of the urinary system | Q60-Q64 |
| MCMs and deformations of the musculoskeletal system | Q65-Q79（Q664–Q666, Q69, Q70 excepted） |
| Other MCMs | Q80-Q89（Q81–Q84 exceped） |
| ICD, international classification of disease; MCM, major congenital malformation | |

| **Supplemental Table 3. List of covariates in this study** | | |
| --- | --- | --- |
| **Covariates** | **Definition** | **Rationale** |
| Maternal age | Maternal age at delivery | Maternal age is a known risk factor for BDs.^1,2^ |
| Delivery year | Calendar year of delivery | This factor partly addresses residual confounding given the long follow-up period of the JDMC claims database. |
| Epilepsy | ICD-10 code: G40, G41 | Epilepsy is considered a maternal disease that can interfere with originally normal child development.^1^ |
| Diabetes | ICD-10 code: E10–E14, O24 | Diabetes has teratogenic effects and significantly increases the risk for major BDs.^1,2^ |
| Obesity | ICD-10 code: E66 | During pregnancy, obesity is associated with an increased risk of BDs.^1^ |
| Teratogenic drugs | Etretinate, carbamazepine, thalidomide, cyclophosphamide, danazol, thiamazole, trimethadione, valproate, vitamin A (retinol), phenytoin, phenobarbital, mycophenolate, misoprostol, methotrexate, and warfarin | Identified based on the Guidelines for Obstetrical Practice in Japan 2020 edition.^3^ |
| BD, birth defect; ICD-10, International Classification of Diseases.  References:  1. Gilbert-Barness E. Teratogenic causes of malformations. Ann Clin Lab Sci. 2010;40(2):99–114.  2. Oliveira CI, Fett-Conte AC. Birth defects: Risk factors and consequences. J Pediatr Genet. 2013;2(2):85–90. doi: 10.3233/PGE-13052.  3. Japan Society of Obstetrics and Gynecology and Japan Association of Obstetricians and Gynecologists. Guidelines for obstetrical practice in Japan 2020 edition. Tokyo: Japan Society of Obstetrics and Gynecology; 2020 (in Japanese). | | |
